# Maternal Polystyrene Nanoplastic Exposure Impairs Cardiac Development in Mouse Offspring and Identifies Lactation as a Sensitive Window in Males

**DOI:** 10.3390/biology15141207

**Published:** 2026-07-22

**Authors:** Xiaorui Zhang, Yingguang Li, Xiaotao Zhang, Wenli Shi, Hui Deng, Lingxian Yi, Shuaizhen Zhou, Daojin Yu

**Affiliations:** 1 Fujian Key Laboratory of Traditional Chinese Veterinary Medicine and Animal Health, College of Animal Sciences, Fujian Agriculture and Forestry University, Fuzhou 350002, China; xiaorui.zhang@sibcb.ac.cn (X.Z.);; 2 Core Facility of Molecular Biology, Center for Excellence in Molecular Cell Science, Shanghai Institute of Biochemistry and Cell Biology, Chinese Academy of Sciences, Shanghai 200031, China

**Keywords:** nanoplastics, maternal exposure, lactational exposure, gut microbiota, cardiac developmental toxicity

## Abstract

Maternal exposure to nanoplastics is a growing concern, but its effects on offspring cardiac development and the relative importance of prenatal and lactational exposure remain unclear. Pregnant C57BL/6J mice were orally exposed to 50 nm polystyrene nanoplastics at 3, 15, or 75 μg/g body weight from gestational day 1 to postnatal day 21. A cross-fostering design was used to distinguish gestational exposure from lactational exposure. Maternal polystyrene nanoplastic exposure caused dose-dependent cardiac dysfunction in offspring, including reduced ejection fraction and fractional shortening, increased myocardial injury markers, cardiomyocyte hypertrophy, and fibrosis. Cross-fostering showed that lactationally exposed offspring exhibited more severe cardiac abnormalities than offspring exposed only during gestation, indicating that the nursing period may represent a more vulnerable window. In male offspring, polystyrene nanoplastic exposure was also associated with gut microbiota dysbiosis and cardiac transcriptomic changes. Enrichment analysis identified downregulation of genes related to AMP-activated protein kinase signalling, and integrated microbiome–transcriptome analysis suggested associations between altered gut taxa and cardiac differentially expressed genes. These findings indicate that maternal polystyrene nanoplastic exposure induces offspring cardiac developmental toxicity, with stronger effects during lactation, and suggest the involvement of gut microbial and cardiac molecular remodelling.

## Simple Summary

Plastic particles are increasingly found in the environment, food, and biological samples, raising concerns about their effects on early-life development. This study examined whether exposure of mother mice to tiny polystyrene plastic particles during pregnancy and nursing affects heart development in their pups. The pups showed weaker heart pumping, increased blood markers of heart injury, enlarged heart muscle cells, and more tissue scarring. By exchanging newborn pups between exposed and unexposed mothers, we found that exposure during nursing produced stronger heart changes than exposure before birth alone. The study also found changes in gut bacteria and heart gene activity, suggesting that disruption of the gut–heart connection may contribute to these effects. These findings highlight nursing as a potentially sensitive period for plastic particle exposure and provide evidence that early-life exposure can affect heart development.

## 1. Introduction

Plastic pollution has evolved into a global environmental crisis, with microplastics and nanoplastics (NPs) being detected in diverse ecosystems and the human food chain [1,2]. Due to their small particle size and high stability, polystyrene nanoplastics (PS-NPs) can easily penetrate biological barriers [3,4]. Recent clinical studies have reported the presence of NPs in the human placenta and breast milk, raising significant concerns regarding the potential developmental toxicity of maternal exposure on the next generation [5,6,7,8].

The perinatal period, encompassing gestation and lactation, is a critical window for organogenesis and systemic development. While previous research has suggested that maternal NP exposure can lead to metabolic disorders and neurotoxicity in offspring, its impact on cardiac development, a process highly sensitive to environmental stressors, remains poorly understood [9,10]. The heart is the first functional organ to develop, and any disturbance during this stage may lead to long-term cardiovascular consequences. However, it remains unclear whether these detrimental effects are more strongly associated with prenatal exposure or lactational exposure. Emerging evidence highlights the gut–heart axis as a potential regulator of cardiovascular health [11]. Environmental pollutants often disrupt the gut microbiota, leading to the translocation of microbial metabolites or the induction of systemic inflammation, which subsequently impairs cardiac function [11,12,13,14]. Serum cTnT, CK-MB, and LDH are commonly used indicators of myocardial injury and are therefore combined with echocardiography and histological evaluation to assess offspring cardiac damage. Firmicutes and Bacteroidota are major bacterial phyla in the mouse gut, and previous studies have shown that plastic particle exposure can disturb the gut microbial composition and intestinal homeostasis. Despite this, the role of the gut microbiota in mediating PS-NP-induced cardiac developmental toxicity via maternal–offspring transmission has not been fully elucidated. Furthermore, the molecular pathways, such as AMP-activated protein kinase (AMPK) signalling, a master regulator of energy metabolism and cellular homeostasis in the heart, warrant deeper investigation in the context of NP exposure [15].

In this study, we established a maternal PS-NP exposure model in C57BL/6 mice to evaluate the dose-dependent effects on offspring cardiac development. By employing a cross-fostering design, we aimed to distinguish the relative contributions of gestational and lactational exposure windows. Furthermore, we integrated 16S rRNA sequencing and cardiac transcriptomic analysis to investigate whether maternal PS-NP exposure was associated with gut microbiota dysbiosis and cardiac molecular alterations in offspring. Our findings provide new evidence for the developmental cardiotoxicity of nanoplastics and highlight the potential importance of the nursing period as a vulnerable window of exposure.

## 2. Materials and Methods

### 2.1. Characterisation of Polystyrene Nanoplastics (PS-NPs)

Polystyrene nanoplastics (PS-NPs, 50 nm in diameter) were purchased from Tomicro (Shanghai, China). The morphology and size distribution of the PS-NPs were characterised by scanning electron microscopy (GeminiSEM 560, Carl Zeiss Microscopy GmbH, Oberkochen, Germany). The hydrodynamic diameter and zeta potential of the PS-NPs dispersed in different media, including deionised water, phosphate-buffered saline (PBS), and Dulbecco’s Modified Eagle Medium (DMEM), were measured using a Zetasizer Nano ZS system (Malvern Panalytical Ltd., Malvern, Worcestershire, UK). The 50 nm particle size was selected to represent nanoscale PS-NPs and to evaluate the potential developmental effects of particles with high biological barrier-interaction potential [16].

### 2.2. Animal Care and Experimental Design

**Figure 1:**
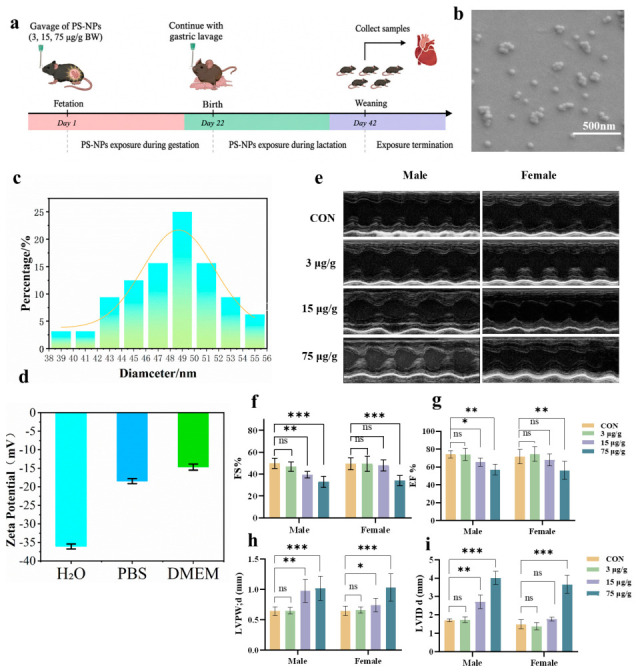
Characterisation of PS-NPs and dose-related cardiac dysfunction in offspring. (**a**) Schematic diagram of maternal exposure experiment. Pregnant dams were orally gavaged with PBS or PS-NPs at 3, 15, or 75 μg/g body weight from gestational day 1 until offspring weaning. (**b**) Representative scanning electron microscopy image of PS-NPs; scale bar, 500 nm. (**c**) Particle-size distribution of PS-NPs. (**d**) Zeta potential of PS-NPs in deionised water, PBS, and DMEM. (**e**) Representative M-mode echocardiograms from male and female offspring. (**f**,**g**) Fractional shortening and ejection fraction. (**h**,**i**) Left-ventricular posterior wall thickness in diastole and left-ventricular internal diameter in diastole. Data are presented as mean ± SD. One-way ANOVA followed by Tukey’s multiple-comparison test was used for comparisons within each sex. * *p* < 0.05, ** *p* < 0.01, *** *p* < 0.001; ns, not significant.

Specific-pathogen-free C57BL/6J mice aged 8 weeks were obtained from Shanghai SLAC Laboratory Animal Co., Ltd. (Shanghai, China; SCXK [Hu] 2022-0004; certificate 20220004095958). The mice were housed in individually ventilated cages with unrestricted access to food and water at 20–25 °C and 40–70% relative humidity under a 12 h light/dark cycle. Noise was maintained at ≤60 dB and illumination at 20 lx. After 1 week of acclimation, the mice were paired at a male-to-female ratio of 1:2. The mice were fed a standard growth diet for rodents throughout the experiment. The diet met the implementation standards GB14924.3-2010 and GB14924.2-2001. The main ingredients included soybean meal, fish meal, chicken meal, brewer’s yeast powder, soybean oil, wheat bran, corn, wheat, vitamins, and mineral supplements. The basic nutrient composition and ingredient information are provided in Figure S2c. The day on which a vaginal plug was detected was designated gestational day 1. In the timelines shown in Figures 1a and 3a, experimental day 22 denoted birth, and experimental day 42 denoted weaning and sample collection [17].

#### 2.2.1. Dose–Response Experiment

Each treatment group was established with three males and six females before mating. Pregnant dams received PBS or PS-NPs at 3, 15 or 75 μg/g body weight once daily by oral gavage from gestational day 1. Gavage continued after parturition until offspring were weaned on experimental day 42. The low, medium and high experimental doses were selected with reference to previous maternal rodent studies [6,10,18]. In this study, the term high-dose group refers to the highest dose evaluated within the experimental dose–response design, namely 75 μg/g body weight. We did not assume environmental equivalence because particle-number exposure depends on the particle size, density, aggregation and analytical recovery. The sample size was selected based on previous maternal PS-NP exposure studies, the need to maintain litter-balanced sampling, and the principle of reducing animal use while retaining sufficient biological replication for group comparisons [6,10,18].

#### 2.2.2. Cross-Fostering Experiment

Cross-fostering was conducted using the high PS-NP dose (75 μg/g body weight). After birth, pups were redistributed into four groups according to prenatal and postnatal exposure: CON (PBS/PBS), TP (PS-NPs/PBS), LP (PBS/PS-NPs), and GP (PS-NPs/PS-NPs). All groups, including CON, underwent the same postnatal handling and regrouping procedure to minimise handling-related bias. Each group comprised five foster dams nursing five independent litters housed individually. Dams received the corresponding treatment once daily by oral gavage from gestational day 1 until weaning. At weaning, two male offspring were randomly selected from each litter, resulting in 10 male offspring per group for downstream sampling. To minimise litter effects, one male offspring from each litter was used for echocardiography, terminal blood collection, and paraformaldehyde-fixed heart tissue collection, yielding five mice per group for cardiac functional, serum biochemical, and histological analyses. Cardiac function was assessed on experimental day 42 by echocardiography. Approximately 500 μL of terminal blood was then collected from the retro-orbital venous plexus, followed immediately by the collection of heart tissues for fixation in 4% paraformaldehyde. The second male offspring from each litter was used for frozen sample collection. Heart tissues and colonic contents from these mice were collected immediately after euthanasia and stored at −80 °C for subsequent RNA-seq, qPCR, and 16S rRNA sequencing analyses. Thus, the major downstream analyses were performed using litter-balanced biological samples, with one offspring per litter for each assay type.

### 2.3. Echocardiography

Cardiac function was assessed on experimental day 42 using a Vevo 2100 high-resolution ultrasound system (FUJIFILM VisualSonics, Inc., Toronto, ON, Canada). Five offspring per group were analysed. The mice were anaesthetised with 1.5% inhaled isoflurane and positioned on a platform maintained at 37 °C. Isoflurane was used because it allows for rapid adjustment of anaesthetic depth and is commonly used for murine echocardiography to maintain stable physiological conditions during short imaging procedures. The heart rate was maintained at 450–550 beats per minute. Left-ventricular M-mode recordings were used to calculate the ejection fraction (EF), fractional shortening (FS), posterior wall thickness in diastole (LVPWd) and internal diameter in diastole (LVIDd). Measurements from three consecutive cardiac cycles were averaged by an investigator blinded to group assignments.

### 2.4. Serum Biochemical Analysis

Blood samples collected as described above were centrifuged at 3000 rpm for 15 min at 4 °C to isolate serum. The concentrations of cardiac injury markers, including cardiac troponin T (cTnT), creatine kinase-MB (CK-MB), and lactate dehydrogenase (LDH), were determined using commercial ELISA kits (Shanghai Enzyme-linked Biotechnology Co., Ltd., Shanghai, China) following the manufacturer’s protocols. These circulating biomarkers were selected because cTnT, CK-MB, and LDH are commonly used indicators of myocardial injury and cardiac toxicity [19].

### 2.5. Histological Examination and Immunofluorescence

Heart tissues were fixed in 4% paraformaldehyde, embedded in paraffin, and sectioned. For H&E and Masson’s trichrome staining, tissue sections were stained with haematoxylin and eosin (H&E) to observe their histological structure and with Masson’s trichrome to evaluate collagen deposition (fibrosis). For the assessment of cardiomyocyte hypertrophy, sections were stained with Wheat Germ Agglutinin (WGA) Alexa Fluor 488 (AAT Bioquest, Inc., Pleasanton, CA, USA). Images were acquired using an Olympus BX51 fluorescence microscope (Olympus Corporation, Tokyo, Japan) and analysed using ImageJ (version 1.54p) software. The cross-sectional areas of at least 100 cardiomyocytes were quantified for each group. For WGA quantification, five paraformaldehyde-fixed hearts per group were analysed, with one offspring selected from each litter. At least 100 cardiomyocytes were measured per group for cardiomyocyte cross-sectional area quantification [20].

### 2.6. 16S rRNA Sequencing and Analysis

Colonic contents from male offspring were collected under sterile conditions on experimental day 42 for gut microbiota analysis. Five biological samples were collected per group, with one mouse randomly selected from each litter to minimise litter effects. Samples were not pooled. Samples were immediately frozen in liquid nitrogen and stored at −80 °C until analysis.

Microbial DNA extraction, library construction, 16S rRNA gene sequencing and initial bioinformatic processing were performed by OE Biotech Co., Ltd. (Shanghai, China). DNA was extracted from colonic contents using a stool microbial DNA extraction kit. The DNA concentration and purity were assessed before PCR amplification. The V3–V4 hypervariable region of the bacterial 16S rRNA gene was amplified, and purified amplicons were pooled in equimolar amounts. Qualified libraries were sequenced in paired-end mode on an Illumina NovaSeq 6000 (Illumina, Inc., San Diego, CA, USA) platform. Raw reads were processed in QIIME 2 (version 2025.4) [21]. Low-quality reads, barcode and adaptor sequences, and chimeric sequences were removed before downstream analysis. Representative sequences were taxonomically assigned against a reference database. The ACE index quantified alpha-diversity richness, and Bray–Curtis dissimilarities quantified beta diversity. Group differences in community composition were tested by permutational multivariate analysis of variance (PERMANOVA). Differentially abundant taxa were identified using DESeq2 (version 1.48.1) with false-discovery-rate correction [22].

### 2.7. Cardiac Transcriptomic Analysis (RNA-Seq)

Total RNA was extracted from left-ventricular tissue using TRIzol (Invitrogen, Carlsbad, CA, USA). Five independent heart samples per group were analysed by RNA sequencing. The RNA concentration and purity were measured using a NanoDrop 2000 (Thermo Fisher Scientific, Waltham, MA, USA), and integrity was assessed using an Agilent 2100 Bioanalyzer (Agilent Technologies, Santa Clara, CA, USA). Samples with an RNA integrity number of at least 7.0 were prepared using the NEBNext Ultra RNA Library Prep Kit for Illumina. Libraries were sequenced on an Illumina NovaSeq 6000 to generate 150 bp paired-end reads. Read quality was inspected using FastQC (version 0.12.1), and adaptors and low-quality bases were removed using Trimmomatic (version 0.39) [23]. Clean reads were aligned to GRCm39/mm39 using HISAT2 (version 2.2.1) [24], and transcripts were assembled and quantified using StringTie (version 3.0.0) [25]. Differential expression was tested using DESeq2 with |log2 fold change| > 1 and adjusted *p* < 0.05. Gene Ontology and KEGG enrichment analyses were performed on the differentially expressed gene sets.

### 2.8. Quantitative Real-Time PCR

Total cardiac RNA was extracted using TRIzol and reverse-transcribed using the PrimeScript RT Reagent Kit (Takara Biomedical Technology (Beijing) Co., Ltd., Beijing, China). Quantitative real-time PCR (qPCR) was performed using TB Green Premix Ex Taq II on a QuantStudio 5 (Thermo Fisher Scientific, Waltham, MA, USA) system. The cycling conditions were 95 °C for 30 s, followed by 40 cycles of 95 °C for 5 s and 60 °C for 30 s. β-actin served as the reference gene, and relative expression was calculated using the 2^−ΔΔCt^ method [26]. Reactions were performed in technical triplicate. Primer sequences are provided in Supplementary Table S1.

### 2.9. Statistical Analysis

Exposure group was considered the independent variable. Echocardiographic parameters, serum biomarkers, the cardiomyocyte cross-sectional area, alpha-diversity indices, and gene-expression values were considered dependent variables. Data are presented as the mean ± SD. Dose–response and cross-fostering endpoints were analysed by one-way ANOVA followed by Tukey’s multiple-comparison test. Dose–response analyses were performed separately by sex. Bray–Curtis community differences were tested by PERMANOVA. Differential microbial taxa and cardiac transcripts were analysed using DESeq2 with false-discovery-rate correction. Spearman correlations were used to relate microbial and transcriptional features. Multi-omics integration was performed using DIABLO in the mixOmics package (version 6.32.0), which implements multiblock sparse partial least-squares discriminant analysis [27,28]. Two-sided *p* < 0.05 was considered statistically significant unless an adjusted-P threshold was specified.

## 3. Results

### 3.1. PS-NP Characterisation and Dose-Related Cardiac Dysfunction in Offspring

Dams received PBS or PS-NPs at 3, 15, or 75 μg/g body weight from gestational day 1 until offspring weaning (Figure 1a). Scanning electron microscopy showed predominantly spherical particles with a nominal diameter of approximately 50 nm and no obvious severe aggregation (Figure 1b). The particle-size distribution was narrow and centred near 50 nm, consistent with the nominal particle size (Figure 1c). The mean zeta potentials were approximately −36 mV in deionised water, −18 mV in PBS, and −15 mV in DMEM, indicating relatively stable dispersion characteristics in the tested media (Figure 1d). No overt growth retardation or marked lethality was observed during the lactation period, as indicated by body weight trajectories and survival curves (Figure S1a–c).

Representative M-mode echocardiograms showed impaired ventricular contractility in offspring exposed to higher PS-NP doses (Figure 1e). In male offspring, fractional shortening and the ejection fraction were lower at 15 and 75 μg/g than in controls (Figure 1f,g). In females, fractional shortening and the ejection fraction were significantly reduced mainly at 75 μg/g, with no significant differences at 3 or 15 μg/g (Figure 1f,g). The left-ventricular posterior wall thickness in diastole was increased at 15 and 75 μg/g in both sexes (Figure 1h). The left-ventricular internal diameter in diastole was increased in males at 15 and 75 μg/g and in females mainly at 75 μg/g (Figure 1i). No echocardiographic variable differed significantly between the 3 μg/g and control groups. Thus, cardiac functional and structural changes occurred at 15 and 75 μg/g in males, whereas comparable changes in females were largely restricted to 75 μg/g.

### 3.2. Maternal PS-NP Exposure Increased Cardiac Injury Markers and Cardiomyocyte Hypertrophy

**Figure 2:**
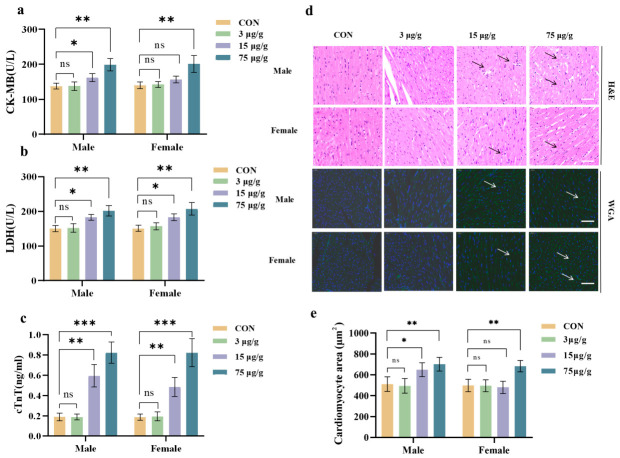
Maternal exposure to PS-NPs increased cardiac injury markers and cardiomyocyte hypertrophy in offspring. (**a**–**c**) Serum CK-MB, LDH, and cTnT levels in male and female offspring. (**d**) Representative H&E- and WGA-stained cardiac sections from male and female offspring. Arrows indicate representative myocardial structural abnormalities or enlarged cardiomyocytes. Scale bars, 50 μm. (**e**) Quantification of cardiomyocyte cross-sectional area based on WGA staining. Data are presented as mean ± SD. One-way ANOVA followed by Tukey’s multiple-comparison test was used for comparisons within each sex. * *p* < 0.05, ** *p* < 0.01, *** *p* < 0.001; ns, not significant.

Serum creatine kinase-MB, lactate dehydrogenase, and cardiac troponin T levels were increased following maternal PS-NP exposure. In male offspring, these myocardial injury markers were significantly elevated mainly at 15 and 75 μg/g, whereas female offspring showed a less pronounced response, with significant changes mainly observed at the higher dose (Figure 2a–c).

Histological analysis further supported the presence of myocardial injury and remodelling. The control myocardium showed aligned fibres and preserved cellular architecture. By contrast, hearts from the 15 and 75 μg/g groups showed enlarged and disorganised cardiomyocytes, widened intercellular spaces, and structural abnormalities, particularly in male offspring (Figure 2d). WGA staining showed larger cardiomyocyte cross-sectional areas after maternal PS-NP exposure (Figure 2d). Quantitative analysis confirmed significant cardiomyocyte hypertrophy in male offspring at 15 and 75 μg/g and in female offspring mainly at 75 μg/g (Figure 2e). No significant difference was detected at 3 μg/g. These results show dose-related increases in serum cardiac injury markers and myocardial abnormalities, with changes occurring at a lower dose in males than in females.

### 3.3. Cardiac Abnormalities Were Concentrated in Groups Exposed During Lactation

**Figure 3:**
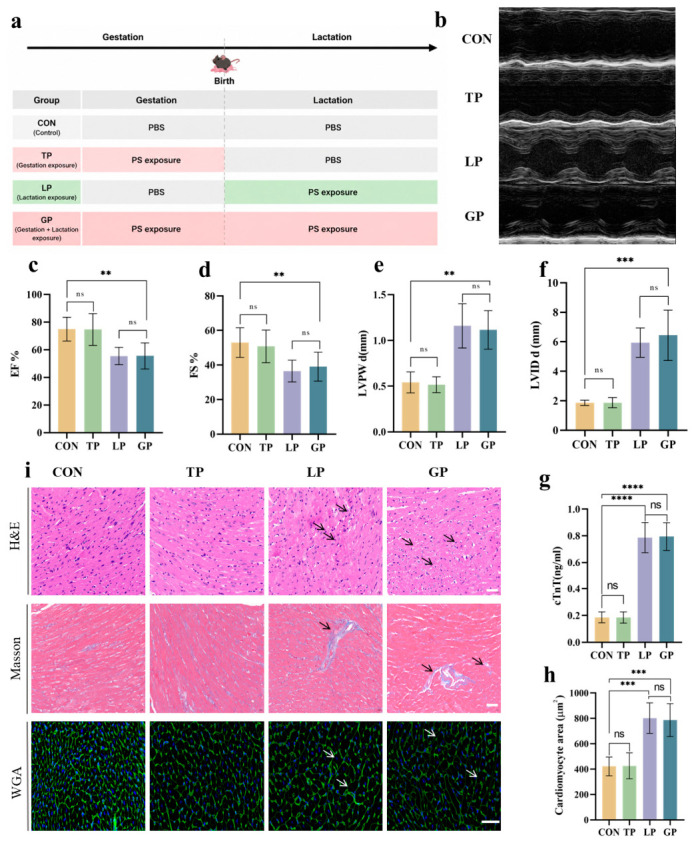
Cardiac abnormalities were concentrated in groups exposed to PS-NPs during lactation. (**a**) Cross-fostering design used to distinguish prenatal and postnatal exposure windows. CON, prenatal and postnatal PBS; TP, prenatal exposure to PS-NPs and postnatal PBS nursing; LP, prenatal PBS and postnatal exposure to PS-NPs; GP, prenatal and postnatal exposure to PS-NPs. (**b**) Representative M-mode echocardiograms from male offspring. (**c**–**f**) Ejection fraction, fractional shortening, left-ventricular posterior wall thickness in diastole, and left-ventricular internal diameter in diastole. (**g**) Serum cTnT level. (**h**) Quantification of cardiomyocyte cross-sectional area. (**i**) Representative H&E-, Masson’s trichrome-, and WGA-stained cardiac sections. Arrows indicate representative myocardial disarray, collagen deposition, or enlarged cardiomyocytes. Scale bars, 50 μm. Data are presented as mean ± SD. One-way ANOVA followed by Tukey’s multiple-comparison test was used. ** *p* < 0.01, *** *p* < 0.001, **** *p* < 0.0001; ns, not significant.

To distinguish the relative contributions of gestational and lactational exposure, a cross-fostering experiment was performed using the high PS-NP dose. Four groups were generated: CON, prenatal and postnatal PBS; TP, prenatal PS-NP exposure and postnatal PBS nursing; LP, prenatal PBS and postnatal PS-NP exposure; and GP, prenatal and postnatal PS-NP exposure (Figure 3a).

Representative M-mode echocardiograms showed more evident cardiac functional alterations in LP and GP offspring, whereas TP offspring were more similar to CON offspring (Figure 3b). Quantitative analysis showed that the ejection fraction and fractional shortening were significantly reduced in LP and GP compared with CON, while TP did not differ significantly from CON (Figure 3c,d). The left-ventricular posterior wall thickness in diastole and left-ventricular internal diameter in diastole were also increased mainly in LP and GP (Figure 3e,f). Serum cardiac troponin T was significantly increased in LP and GP, whereas the level in TP remained close to the control level (Figure 3g). Similar patterns were observed for CK-MB and LDH (Figure S2a,b).

Histological analysis further supported this exposure-window pattern. H&E staining showed disrupted myocardial architecture mainly in LP and GP. These groups also showed increased collagen deposition in Masson-stained sections and enlarged cardiomyocytes in WGA-stained sections (Figure 3i). Quantitative WGA analysis confirmed a significant increase in cardiomyocyte cross-sectional area in LP and GP, with no significant difference between LP and GP (Figure 3h). Together, these results indicate that lactational-only and continuous exposure produced comparable cardiac abnormalities, whereas gestational-only exposure showed no detectable effect on the measured endpoints.

### 3.4. Gut Microbial Diversity and Composition Differed Across Exposure Windows

**Figure 4:**
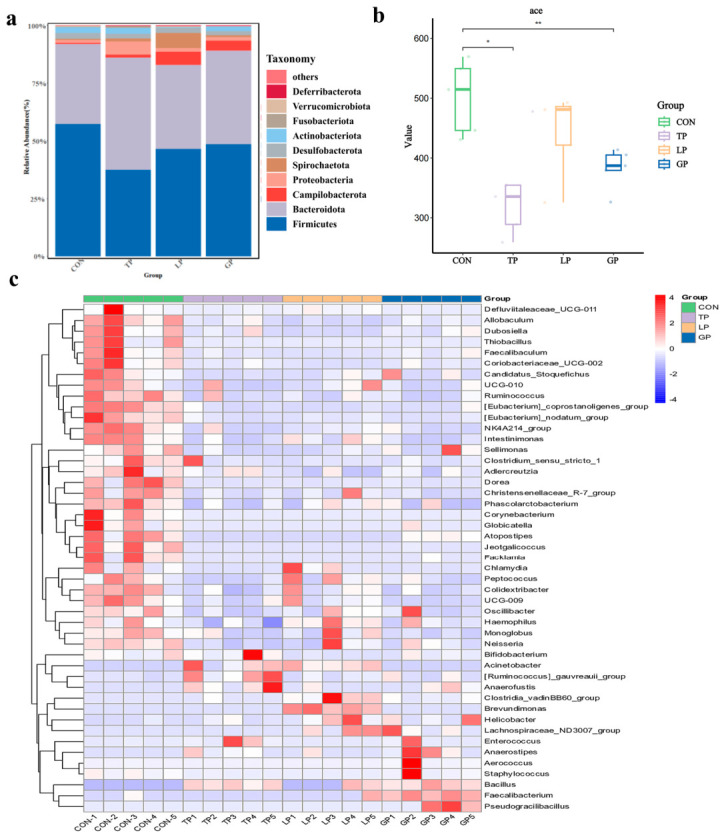
Gut microbial diversity and composition differed across PS-NP exposure windows in male offspring. (**a**) Relative abundance of gut microbiota at the phylum level in the CON, TP, LP, and GP groups. (**b**) Alpha diversity measured by the ACE index. (**c**) A genus-level hierarchical clustering heatmap showing microbial abundance patterns across the four groups. Data are presented as the mean ± SD where applicable. One-way ANOVA followed by Tukey’s multiple-comparison test was used for ACE index comparisons. * *p* < 0.05, ** *p* < 0.01.

To evaluate whether maternal PS-NP exposure altered the gut microbial composition, 16S rRNA sequencing was performed using colonic-content samples from male offspring in the CON, TP, LP, and GP groups. At the phylum level, Firmicutes and Bacteroidota were the dominant bacterial phyla across all groups, but their relative abundances varied among the four exposure-window groups (Figure 4a). Alpha-diversity analysis showed that the ACE index was significantly reduced in TP and GP compared with CON, indicating decreased microbial richness following maternal PS-NP exposure (Figure 4b). Genus-level hierarchical clustering further showed distinct microbial abundance patterns across the CON, TP, LP, and GP groups (Figure 4c).

Additional microbiota analyses are shown in Figure S3. The flower plot shows the microbial features shared by all four groups, with additional features detected in individual samples or groups (Figure S3a). Bray–Curtis principal-coordinate analysis showed separation of microbial community structures among the groups, with significant group differences by PERMANOVA (*p* = 0.001, R^2^ = 0.282, Figure S3b). NMDS analysis showed a similar pattern of intergroup variation (Figure S3c). Differential abundance heatmaps and species-level abundance analysis further indicated exposure-window-associated microbial alterations (Figure S3d,e). These results suggest that maternal PS-NP exposure disturbed the gut microbial richness and composition in male offspring.

### 3.5. Cardiac Transcriptional Responses Varied by Exposure Window

**Figure 5:**
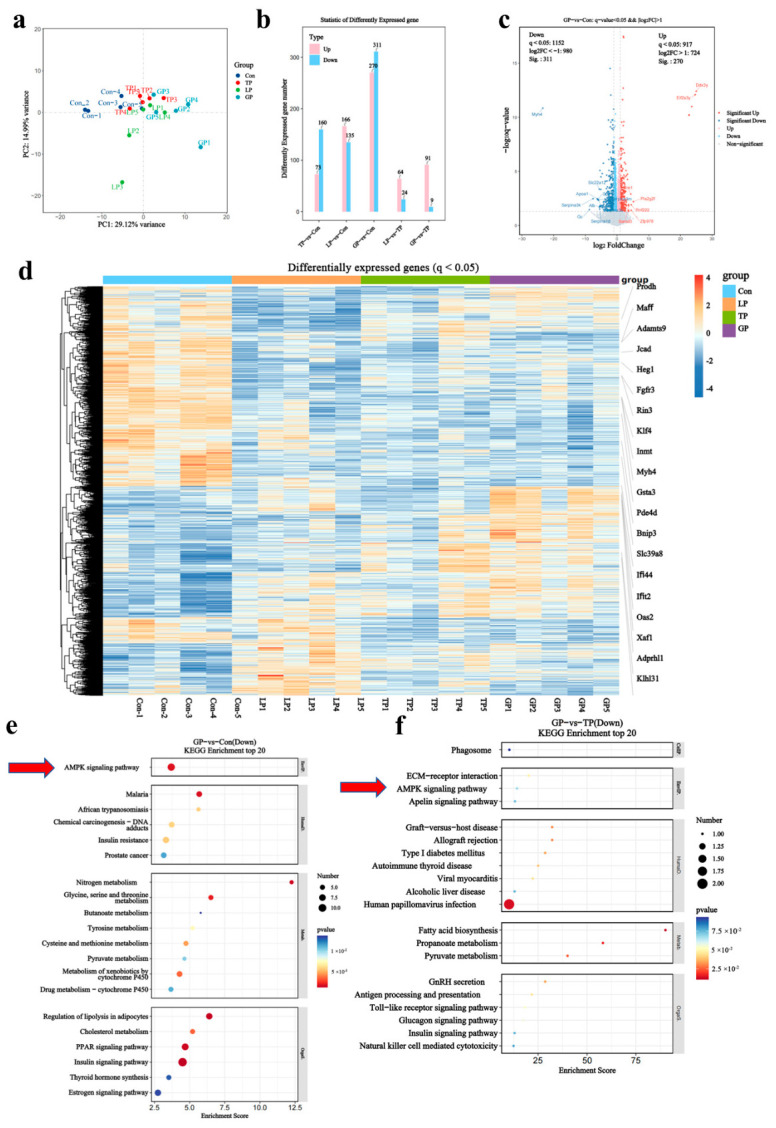
Cardiac transcriptional responses varied by exposure window in male offspring. (**a**) Principal-component analysis of cardiac RNA-seq profiles from male offspring in the CON, TP, LP, and GP groups. (**b**) The numbers of upregulated and downregulated differentially expressed genes in the indicated comparisons. (**c**) A volcano plot of differentially expressed genes in the GP versus CON comparison. Red dots indicate significantly upregulated genes, blue dots indicate significantly downregulated genes, and grey dots indicate non-significant genes. (**d**) A hierarchical clustering heatmap of differentially expressed genes among the four groups. (**e**,**f**) KEGG enrichment analysis of downregulated genes in the GP versus CON and GP versus TP comparisons, respectively. The AMPK signalling pathway is indicated by red arrows. Differential expression was defined as adjusted *p* < 0.05 and |log2 fold change| > 1.

Principal-component analysis of cardiac RNA sequencing profiles separated CON from exposed samples along PC1 and PC2, which explained 29.12% and 14.09% of the variance, respectively. LP samples showed greater within-group dispersion than did the other groups (Figure 5a). Differential expression analysis identified exposure-window-specific transcriptional changes. Relative to CON, TP contained 73 upregulated and 160 downregulated genes, LP contained 166 upregulated and 135 downregulated genes, and GP contained 270 upregulated and 311 downregulated genes. In addition, the LP-versus-TP comparison contained 64 upregulated and 24 downregulated genes, whereas the GP-versus-TP comparison contained 91 upregulated and nine downregulated genes (adjusted *p* < 0.05 and |log2 fold change| > 1; Figure 5b).

The volcano plot for GP versus CON showed extensive transcriptional alterations, including both significantly upregulated and downregulated genes (Figure 5c). Hierarchical clustering of genes with adjusted *p* < 0.05 separated the four groups and showed distinct expression patterns in TP, LP, and GP relative to CON (Figure 5d). Genes downregulated in GP relative to CON were enriched in AMPK signalling, insulin resistance, PPAR signalling, insulin signalling, cholesterol metabolism, and regulation of lipolysis (Figure 5e). Genes downregulated in GP relative to TP were enriched in AMPK signalling, extracellular-matrix receptor interaction, apelin signalling, phagosome, fatty-acid biosynthesis, pyruvate metabolism, and immune-related pathways (Figure 5f). AMPK signalling occurred in both enriched downregulated-gene sets. Thus, each exposure window produced a distinct cardiac transcriptional profile, with GP showing the largest number of differentially expressed genes.

### 3.6. Microbial and Cardiac Transcriptional Features Covaried Across Exposure Contrasts

DIABLO integration yielded correlations of 0.94 between selected microbial and RNA components for GP versus TP and 0.97 for TP versus LP. The corresponding component score plots separated the compared groups (Figure S4a,b). Spearman correlation heatmaps showed positive and negative associations between selected microbial taxa and cardiac genes in the GP-versus-TP and TP-versus-LP contrasts (Figure S4c,d).

Quantitative PCR showed lower Pdk4, Pfkfb2, and Slc2a1 expression and higher Ucp3 expression in LP and GP than in CON, with no significant TP–CON difference (Figure S4e–h). LP and GP showed the same direction of change for all four genes, consistent with the RNA sequencing profiles. These results suggest a potential association between gut microbial dysbiosis and cardiac metabolic transcriptional alterations, although the present analysis remains correlative.

## 4. Discussion

This study distinguished cardiac effects associated with gestational and lactational maternal PS-NP exposure. Dose–response exposure produced functional, biochemical and structural abnormalities in offspring hearts at weaning, with several changes occurring at a lower dose in males than in females. Cross-fostering showed comparable cardiac phenotypes in LP and GP, whereas TP did not differ from CON for the measured endpoints. These findings identify lactation as a sensitive exposure window in male offspring in this mouse model and suggest that gut microbial alterations may be associated with cardiac transcriptional changes.

The cardiac phenotype is consistent with evidence that PS-NPs can disrupt myocardial redox balance, inflammatory signalling and energy metabolism. Respiratory exposure to 40 nm PS-NPs caused dose- and time-dependent cardiac injury, systolic dysfunction and mitochondrial damage in mice [29]. Oral exposure also produced chamber enlargement and reduced contractile function, accompanied by TNF-α/NF-κB and p38/MAPK activation, oxidative stress, fibrosis and apoptosis [30]. These processes offer plausible routes from PS-NP exposure to the increased injury markers, collagen staining and ventricular remodelling observed here. However, we did not measure reactive oxygen species, inflammatory mediators, mitochondrial function or pathway activation.

The sex pattern also extends earlier observations. Maternal PS-NP exposure has been reported to induce sex-specific cardiac transcriptional changes, macrophage infiltration, fibrosis and apoptosis in offspring [31]. Here, males showed significant systolic and morphometric changes at 15 μg/g, whereas several corresponding female endpoints changed only at 75 μg/g. Therefore, male offspring were selected for the initial cross-fostering-related tissue collection and molecular analyses because they showed detectable cardiac changes at a lower exposure dose and were considered more suitable for exploring exposure-window-associated mechanisms in this study. Sex-dependent placental responses, particle disposition, endocrine signalling or cardiac maturation may contribute to this difference. These possibilities require direct testing because molecular analyses in the cross-fostering experiment were limited to male offspring, and future studies should include sex-stratified multi-omics analyses to determine whether similar mechanisms occur in female offspring.

Several features could make lactation more sensitive than gestation. The neonatal intestinal barrier and gut microbiota are still developing, and postnatal exposure may alter epithelial integrity, microbial metabolites and systemic immune signals during cardiac metabolic maturation. Quantitative radiolabelling in pregnant rats showed that 20 nm PS-NPs were transferred through both placenta and milk, whereas 100 nm particles were detected only in milk [32]. A separate mouse study detected fluorescent PS-NPs in maternal mammary glands and in offspring intestine and liver after maternal drinking-water exposure during lactation, together with intestinal barrier disruption [33]. These studies support the plausibility of lactational particle transfer but do not establish the route in our model. Cross-fostering also exchanges maternal care, nursing-dam microbiota, milk composition, and the postnatal environment, and cross-fostering itself can induce durable microbiota shifts [34]. Therefore, the similarity between the LP and GP groups suggests a possible contribution of lactational exposure, but it does not establish milk-borne particle transfer as the underlying mechanism.

The multi-omic results are compatible with, but do not establish, a microbiota–metabolism link to the heart. LP and GP showed concordant reductions in Pdk4, Pfkfb2 and Slc2a1 and increased Ucp3 expression. These genes participate in substrate selection, glucose utilisation and mitochondrial coupling [15,35,36,37]. AMPK signalling was enriched among genes downregulated in GP relative to CON and TP, providing a transcriptional context for altered myocardial energy handling. PS-NPs can disrupt the intestinal barrier and microbiota in adult mice [38], and gut-derived metabolites have been linked to cardiovascular physiology [11,39,40,41]. The microbial community structure differed across exposure windows, and integrated microbiome–transcriptome analysis suggested potential associations between microbial alterations and cardiac transcriptional changes.

The findings are relevant to environmental exposure during pregnancy and breastfeeding but do not quantify human risk. Microplastics have been detected in human placenta, meconium and breast milk [5,8], and experimental studies have shown the placental transfer and foetal distribution of nanoscale polystyrene [3,4,42,43]. In adults with carotid atheroma, detected micro- and nanoplastics were associated with subsequent cardiovascular events. Residual confounding and the observational design precluded causal inference [44]. Translation of our mouse findings is limited by the uncertain environmental equivalence of the doses; male-only molecular profiling; absence of particle measurements in maternal blood, placenta, milk and offspring tissues; and assessment at one postnatal time point. Future animal studies should use litter-aware designs, quantify particle transfer, distinguish transient from persistent cardiac effects and experimentally test the putative microbiota–AMPK-related pathway. Prospective maternal–infant studies should combine contamination-controlled particle measurements with exposure-source information and longitudinal cardiovascular assessment. Such evidence is needed before maternal PS-NP exposure can be linked to clinically meaningful developmental cardiac risk.

## 5. Conclusions

Maternal PS-NP exposure impaired offspring cardiac function and structure, with the clearest dose–response effects observed at 15 and 75 μg/g. Cross-fostering identified lactation as a sensitive postnatal exposure window. These findings provide new evidence that maternal nanoplastic exposure disrupts cardiac development and may involve gut microbiota–cardiac metabolic alterations, highlighting the importance of considering lactational exposure when evaluating the developmental health risks of environmental nanoplastics.

## Data Availability

The raw RNA-seq and 16S rRNA sequencing data generated in this study are being deposited in a public repository. Other data supporting the findings of this study are available from the corresponding author upon reasonable request.

## Supplementary Materials

The following supporting information can be downloaded at https://www.mdpi.com/article/10.3390/biology15141207/s1, Figure S1: Body weight trajectories and survival of offspring during the lactation period. Figure S2: Additional myocardial injury markers in male offspring from the cross-fostering experiment and diet composition information. Figure S3: Supplementary gut microbiota analyses in male offspring. Figure S4: Integrated microbiome–transcriptome associations and qPCR validation in male offspring. Table S1: Primers used in this study.
