# Supplementary material for: Maternal Polystyrene Nanoplastic Exposure Impairs Cardiac Development in Mouse Offspring and Identifies Lactation as a Sensitive Window in Males
Source: Biology (Basel). 2026 Jul 22;15(14):1207. doi: 10.3390/biology15141207 (PMC13403680; doi:10.3390/biology15141207)
Supplement: Supplementary file 1 [file biology-15-01207-s001.zip › Supplementary File.pdf]

Figure S1. Body weight trajectories and survival of offspring during the lactation period.

(a) Body weight changes of male offspring from postnatal day 3 to postnatal day 21. (b) Body weight changes of female offspring from postnatal day 3 to postnatal day 21. (c) Kaplan–Meier survival curves of offspring during the lactation period. Data in (a,b) are presented as mean ± SD.

Figure S2. Additional myocardial injury markers in male offspring from the cross-fostering experiment and diet composition information.

(a,b) Serum CK-MB and LDH levels in male offspring from the CON, TP, LP, and GP groups. Data are presented as mean ± SD. One-way ANOVA followed by Tukey's multiple-comparison test was used. ***P* < 0.01, ****P* < 0.001; ns, not significant. (c) Ingredient sources and nutrient composition of the standard growth diet for rodents used in this study, according to the manufacturer's certificate of analysis.

Figure S3. Supplementary gut microbiota analyses in male offspring.

(a) Flower plot showing shared and sample-specific microbial features among CON, TP, LP, and GP groups. (b) Principal-coordinate analysis based on Bray–Curtis distance showing differences in microbial community structure among groups. The PERMANOVA result is shown as $R^2 = 0.282$, P = 0.001. (c) Three-dimensional NMDS plot showing intergroup differences in microbial community structure. (d) Heatmap of differential abundance at the phylum level. (e) Relative abundance of significantly altered taxa at the species level.

Figure S4. Integrated microbiome–transcriptome associations and qPCR validation in male offspring.

(a,b) DIABLO component correlations between selected microbial and RNA features in the GP versus TP comparison (a, r = 0.94) and the TP versus LP comparison (b, r = 0.97). (c,d) Spearman correlation heatmaps showing associations between selected microbial taxa and cardiac genes in the GP versus TP and TP versus LP comparisons, respectively. (e–h) qPCR validation of Pdk4, Ucp3, Pfkfb2, and Slc2a1 expression in cardiac tissues. Data in (e–h) are presented as mean ± SD. One-way ANOVA followed by Tukey's multiple-comparison test was used for qPCR comparisons. ****P* < 0.001, *****P* < 0.0001; ns, not significant.
