# Supplementary material for: Maternal Polystyrene Nanoplastic Exposure Impairs Cardiac Development in Mouse Offspring and Identifies Lactation as a Sensitive Window in Males
Source: Biology (Basel). 2026 Jul 22;15(14):1207. doi: 10.3390/biology15141207 (PMC13403680; doi:10.3390/biology15141207)
Supplement: Supplementary file 1 [file biology-15-01207-s001.zip › Table S1.pdf]

Table S1 Primers used in this study

Primer	primer sequence(5’ →3’)	application
RT-Ucp3-F	CAACTGTGCTGAGATGGTGACC	Real-time PCR
RT-Ucp3-R	TGGCACAGAAGCCAGCTCCAAA	Real-time PCR
RT-Pdk4-F	GTCGAGCATCAAGAAAACCGTCC	Real-time PCR
RT-Pdk4-R	GCGGTCAGTAATCCTCAGAGGA	Real-time PCR
RT-Pfkfb2-F	CGAGAGCGAGTTCAACCTTTTGG	Real-time PCR
RT-Pfkfb2-R	CTTCAACTGGCTCGTCCACACT	Real-time PCR
RT-Slc2a1-F	GCTTCTCCAACTGGACCTCAAAC	Real-time PCR
RT-Slc2a1-R	ACGAGGAGCACCGTGAAGATGA	Real-time PCR
β actin-F	CATTGCTGACAGGATGCAGAAGG	Real-time PCR
β actin-R	TGCTGGAAGGTGGACAGTGAGG	Real-time PCR
