# Supplementary figures and images for: Maternal Polystyrene Nanoplastic Exposure Impairs Cardiac Development in Mouse Offspring and Identifies Lactation as a Sensitive Window in Males
Source: Biology (Basel). 2026 Jul 22;15(14):1207. doi: 10.3390/biology15141207 (PMC13403680; doi:10.3390/biology15141207)

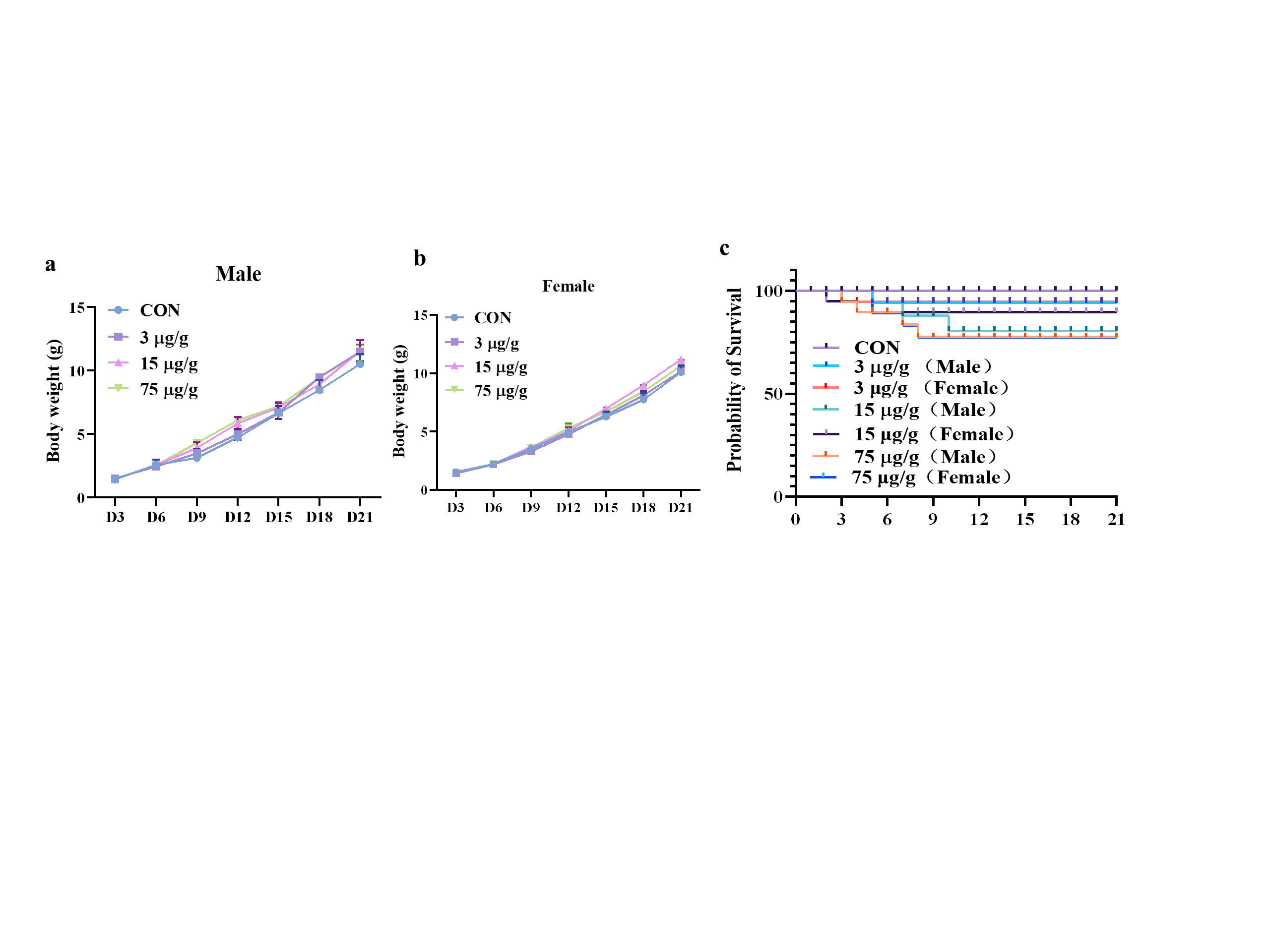

Supplement: Supplementary file 1 [file biology-15-01207-s001.zip › Figure S1.tiff]

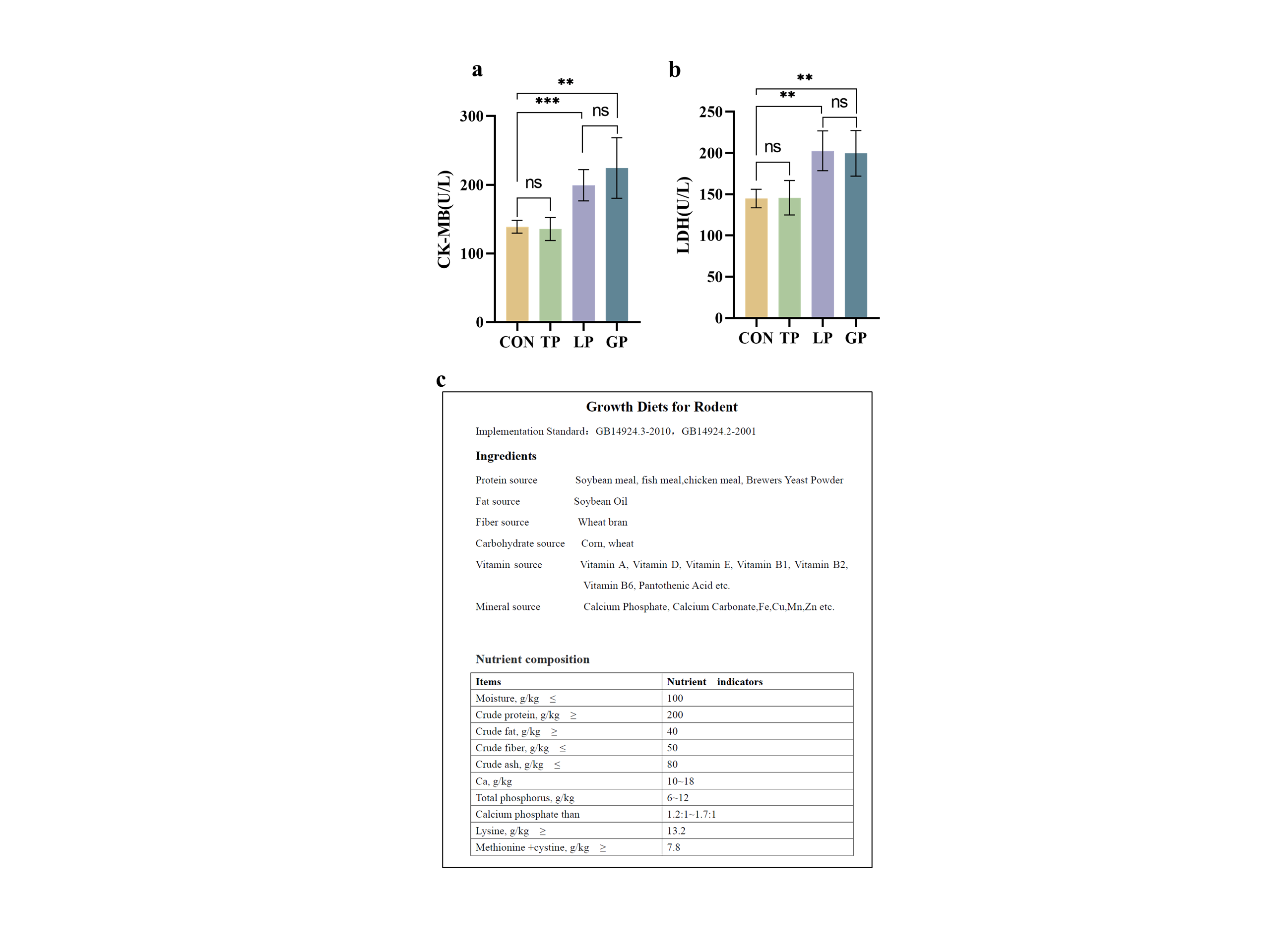

Supplement: Supplementary file 1 [file biology-15-01207-s001.zip › Figure S2.tiff]

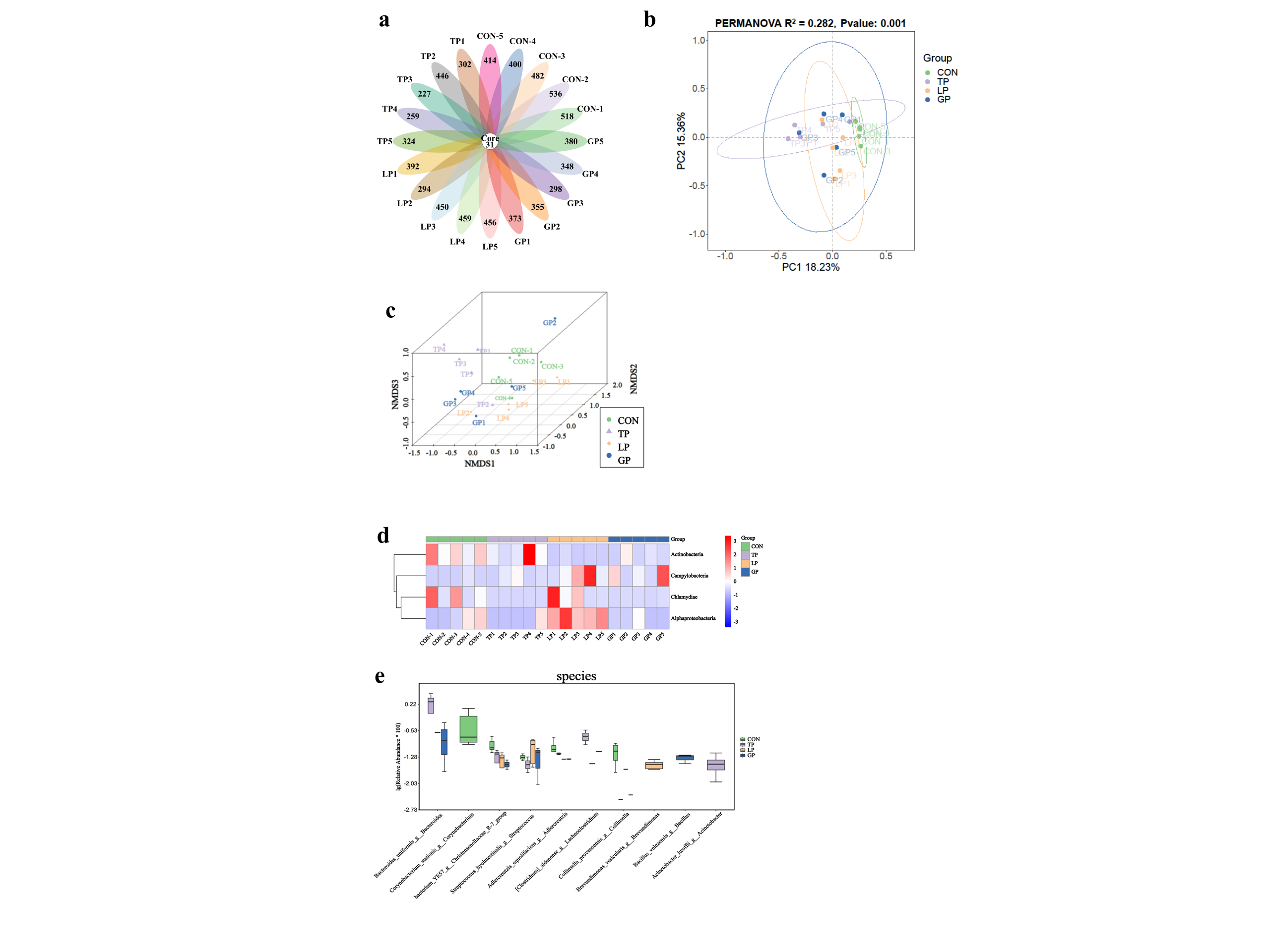

Supplement: Supplementary file 1 [file biology-15-01207-s001.zip › Figure S3.tiff]

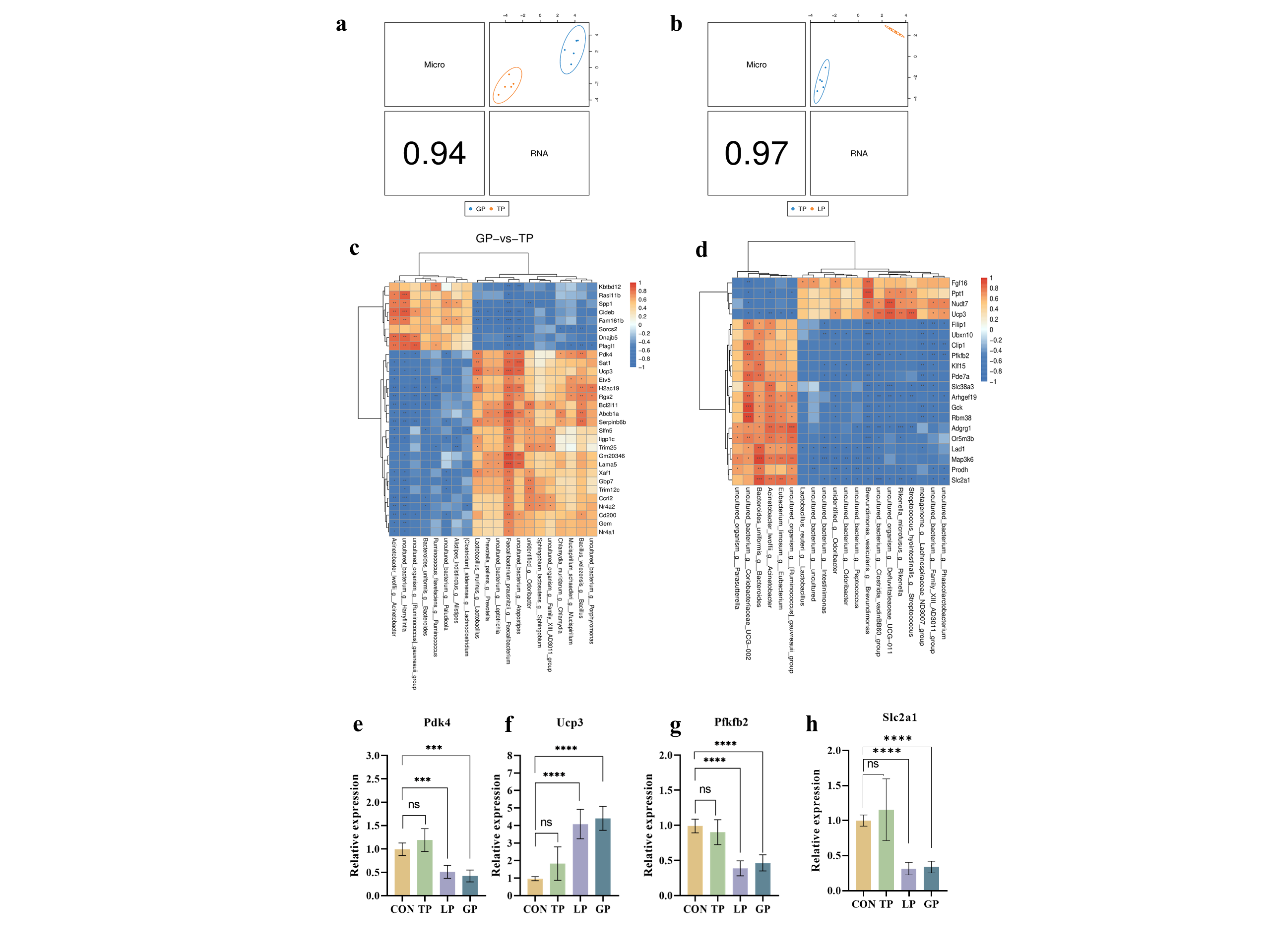

Supplement: Supplementary file 1 [file biology-15-01207-s001.zip › Figure S4.tiff]
